# Coping self-efficacy and personal growth in the situation of the COVID-19 threat

**DOI:** 10.1192/j.eurpsy.2024.1080

**Published:** 2024-08-27

**Authors:** O. G. Kvasova, E. A. Karacheva, P. I. Prichkodko, M. S. Magomed-Eminov, O. O. Savina

**Affiliations:** ^1^Psychological Helping and resocialization Department; ^2^Head of the Psychological Helping and resocialization Department, Moscow State University after M.V.Lomonosov, Moscow, Russian Federation

## Abstract

**Introduction:**

In studies of the socio-psychological consequences of the COVID-19 pandemic, models focused on the negative aspects of stressors, dysfunctions,  anxiety We present the attempt to expand the context and include in the field positive personal resources for psychological well-being and even post traumatic personality growth after disasters. Sometimes separation from family and friends, lack of medicines and medical resources, loss of income, social isolation to humanity, do not automatically assume that a person is capable and responsible for effectively coping with life difficulties.

**Objectives:**

397 (students and patients of clinic average age 26, 2/3 are female

**Methods:**

Peritraumatic Distress Index (CPDI) (Feng, 2020); Impact of Event Scale (Horowitz, 1979), Coping Self-efficacy Scale (Chespeu et al, 2006); Posttraumatic Growth Inventory (Tedeschi& Calhoun, 1996), MMI (Nuttin, 1986) – adapted by M. Magomed-Eminov.

**Results:**

Significant negative correlation between coping self-efficacy and intensity of the impact of stressful events (IES) (rS = - 0,140, p < 0,05) was predictable.

CPDI and PTG showed significant correlation (Pearson’s r= 0.23, p<0.01) between Peritraumatic Distress Index and Post-Traumatic Growth indicators only in the group of respondents who have had  COVID-19. The data is confirmed by the content analysis of incomplete sentences of the subjects of COVID group. The correlation between these indicators in the Non-Covid group was insignificant.

Moreover additional information we got from narratives of infected patients: the data has been split into 3 groups of narratives in the context of cultural-historical activity theory which shows the triadic outcome of survivor after trauma : a) suffering, b) adaptation, coping, resilience, c) personal growth.

**Conclusions:**

To interpret the data the authors propose the meaning-activity approach and personality work with negative life experience (Magomed-Eminov, 1998, 2007, 2009, 2021). Authors suggest that further research on the positive consequences of stressful events beside coping styles and mechanisms that would expand the repertoire of tools predicted the ability of a modern person to cope with adversity and use experience for deeper involvement of human resources with the help of personality work with personal experience.

**Disclosure of Interest:**

None Declared

